# Estimating the Frequency of Lyme Disease Diagnoses, United States, 2010–2018

**DOI:** 10.3201/eid2702.202731

**Published:** 2021-02

**Authors:** Kiersten J. Kugeler, Amy M. Schwartz, Mark J. Delorey, Paul S. Mead, Alison F. Hinckley

**Affiliations:** Centers for Disease Control and Prevention, Fort Collins, Colorado, USA

**Keywords:** Lyme disease, Borrelia burgdorferi, bacteria, diagnoses, insurance claims, disease burden, vector-borne infections, United States

## Abstract

By using commercial insurance claims data, we estimated that Lyme disease was diagnosed and treated in ≈476,000 patients in the United States annually during 2010–2018. Our results underscore the need for accurate diagnosis and improved prevention.

Lyme disease is caused by *Borrelia burgdorferi* spirochetes, which are transmitted to humans by certain *Ixodes* spp. ticks (*1*). The infection can involve multiple organ systems and is treatable with antimicrobial drugs; most persons recover fully, especially those who receive early and appropriate treatment (*1*). The geographic distribution of Lyme disease in the United States and the demographic characteristics of persons affected have been well documented through nearly 3 decades of public health surveillance (*2*). 

However, the frequency of Lyme disease is less well understood. Although 30,000–40,000 cases are reported through surveillance each year, substantial underreporting occurs, as is typical for passively reported surveillance data (*1*). A previous analysis of insurance claims data for the years 2005–2010 estimated that Lyme disease was diagnosed in ≈329,000 persons annually in the United States (*3*). We use similar methods to develop an estimate for 2010–2018.

## The Study

The IBM Watson Health MarketScan Commercial Claims and Encounters Databases (https://www.ibm.com/products/marketscan-research-databases) are derived from insurance claims for inpatient, outpatient, and prescription services covering >25 million privately insured US residents <65 years of age. As detailed elsewhere, we identified Lyme disease diagnoses among the MarketScan population during 2010–2018 by linking specific billing codes for patient encounters with antimicrobial prescriptions (*4*). An outpatient Lyme disease diagnosis was identified by an International Classification of Diseases, 9th Revision, Clinical Modification (ICD-9-CM) or International Classification of Diseases, 10th Revision, Clinical Modification (ICD-10-CM) code for Lyme disease (ICD-9-CM: 088.81; ICD-10-CM: A69.2x) combined with an associated prescription of >7 days’ duration for an appropriate antibiotic drug (*3**,**4*). Inpatient diagnoses were identified according to primary and secondary Lyme disease diagnosis codes (*4*). To minimize the influence of nonincident diagnoses, we excluded any events that occurred in the same person in subsequent years. Age, sex, geographic distribution, and seasonality of Lyme disease diagnoses in MarketScan during 2010–2018 are reported elsewhere (*4*).

To enable extrapolation of rates from the commercially insured population to the US population, we calculated directly standardized case counts according to 5-year age group and state using US Census Bureau 2015 population estimates. Because MarketScan does not include patients >65 years of age, we multiplied the sum of these counts by a factor derived from contemporaneous surveillance data (https://wwwn.cdc.gov/nndss) (Figure). Among confirmed and probable Lyme disease cases reported during 2010–2018, 80.3% were among persons <65 years of age. Thus, we multiplied the standardized case count by 1/0.803, or ≈1.25, to estimate the number of persons of all ages coded and treated for Lyme disease.

**Figure:**
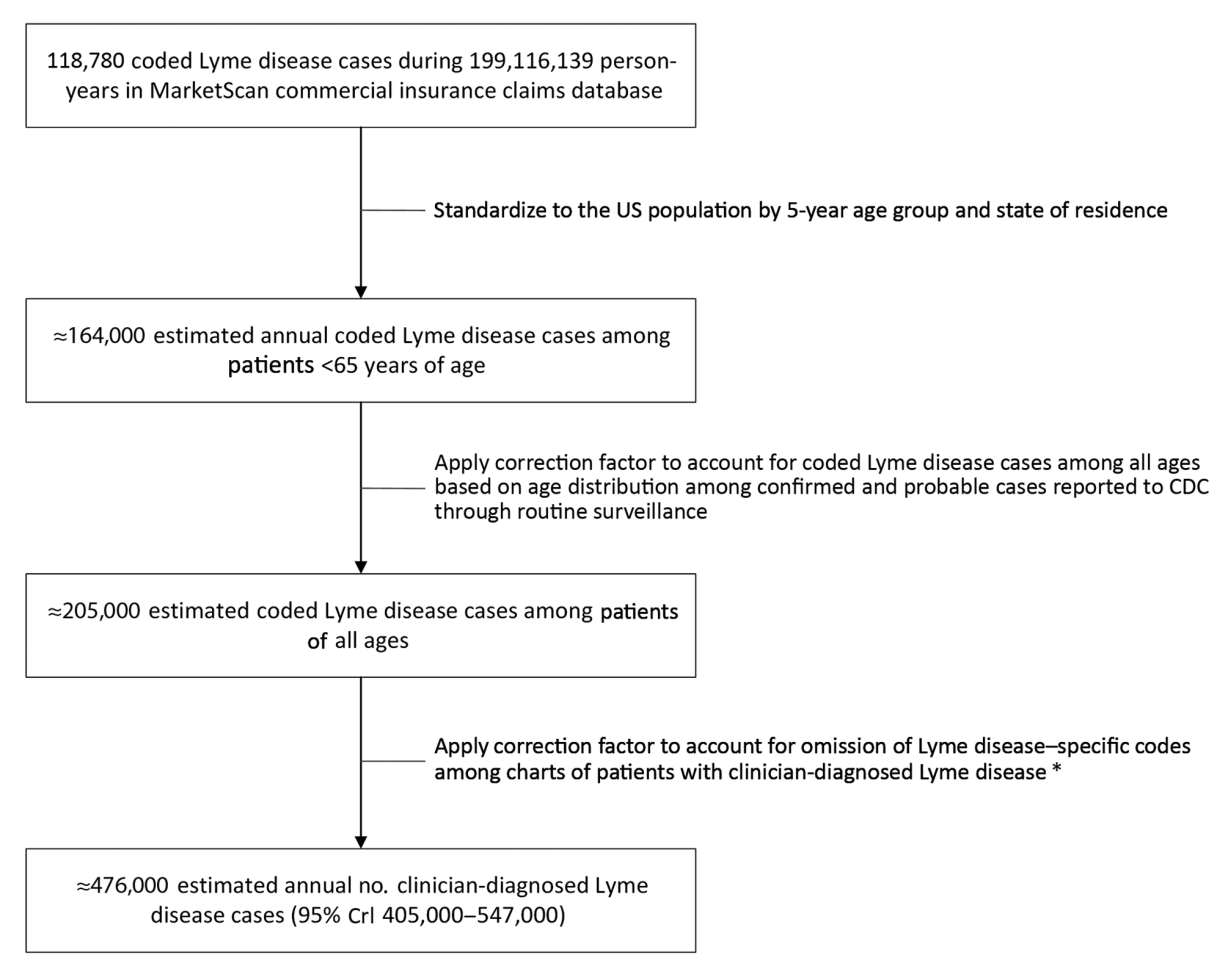
Estimated number of Lyme disease diagnoses annually, calculated by using commercial insurance claims data and 3 correction factors, United States, 2010–2018. Only those records that contained age and state information were included to enable calculation of standardized case counts for the US population. *Correction factor accounting for Lyme disease–specific codes is based on data from 3 studies that suggest only 43% of patients whose cases met the confirmed, probable, or suspect surveillance case definitions had the ICD-9-CM code for Lyme disease in their medical records (*5–7*; E. Schiffman, Minnesota Department of Health, pers. comm., 2020 Jan 17). CDC, Centers for Disease Control and Prevention; CrI, credible interval; ICD-9-CM, International Classification of Diseases, 9th Revision, Clinical Modification.

Previous research has demonstrated that medical records of patients with Lyme disease frequently lack the specific ICD-9 code for the condition (*5**,**6*). To adjust for this undercoding of medical records, we applied a correction factor by using data from 3 studies on the proportion of medical records that contain the ICD-9 code 088.81 and meet the confirmed, probable, and suspect Lyme disease surveillance case definitions (as a proxy for clinician diagnosis). In New York, 114 (41.8%) of 273 records meeting these definitions contained 088.81 (*6*). In Maryland, 84 (35.6%) of 236 records contained 088.81 (*5*). Supplemental analysis of data from Minnesota captured as previously described (*7*) revealed that 91 (56%) of 163 charts contained 088.81 (E. Schiffman, Minnesota Department of Health, pers. comm., 2020 Jan 17). A total of 289 (43.0%) of 672 Lyme disease patients had 088.81 in their medical records. Thus, we multiplied the standardized and age-corrected number of cases by 1/0.430 or ≈2.33 to arrive at an estimate of the frequency of clinician-diagnosed Lyme disease (Figure). A 95% credible interval for this estimate was calculated as previously described (*3*).

A total of 118,780 persons with the requisite codes for Lyme disease were identified in MarketScan among 199,116,139 person-years of observation during 2010–2018. Overall, 81% of these diagnoses occurred among residents of 14 high-incidence states in the Northeast, mid-Atlantic, and upper Midwest; another 8% occurred among residents of adjoining states. After direct standardization and age correction, we found that an average of 205,000 patients were coded and treated for Lyme disease annually. Upon further correction for omission of Lyme disease–specific codes in patient records, we estimate an average of ≈476,000 patients received a diagnosis of Lyme disease each year (95% credible interval 405,000–547,000) during 2010–2018 (Figure).

## Conclusions

The public health burden of an infectious disease can be quantified in several ways: these include the number of illnesses meeting a specific definition that are reported to public health officials; the total number of actual infections resulting in illness in the community; or the number of patients in whom the presumed illness is diagnosed and treated, regardless of actual infection. Our estimate addresses the last of these; it reflects the overall societal and clinical burden of Lyme disease.

We estimate that ≈476,000 persons were diagnosed with Lyme disease annually during 2010–2018. This figure is greater than an estimate of ≈329,000 annual diagnoses for the period 2005–2010. Although both estimates were calculated by using similar methods, we implemented a slightly more restrictive approach that prohibited any patient with a diagnosis of Lyme disease from being counted more than once during the 9-year study period (*3*). The observed increase in Lyme disease diagnoses between these 2 periods parallels increases in cases reported through surveillance (*1*).

Our estimate is based on commercial insurance claims data that might not be representative of the US population with respect to disease risk and access to health care. In addition, the correction factor used to account for omission of Lyme disease–specific ICD-9-CM and ICD-10-CM codes in medical records is based on a review of codes in only 672 medical records, yet it more than doubles the estimated number of diagnoses. Without this correction factor, the observed rate of diagnoses in our study would be similar to the 76 diagnoses/100,000 persons per year reported by Tseng et al. (*8*) in a separate analysis of claims data. Further studies of coding patterns and improved access to and use of electronic health records could fill these data gaps, enabling more robust and precise estimates in the future (*9*).

The estimates we report are influenced by the uncertainties of clinical practice, in which patients are often treated presumptively, inevitably resulting in some degree of overdiagnosis and overtreatment (*10*). In contrast, cases reported through national Lyme disease surveillance meet a standardized case definition and are more likely to represent actual infections. However, routine surveillance is subject to substantial underreporting, previously estimated at between 3- and 12-fold for Lyme disease (*1*). The difference between our estimate and the ≈35,000 cases reported annually though surveillance is a result of the combined effects of underreporting of infections and overdiagnosis in clinical practice. Our analysis does not enable us to discern the relative contribution of each. Although we implemented restrictions to mitigate inclusion of retreatment for nonincident diagnoses, overdiagnosis could account for the proportionally higher number of diagnoses in residents of low-incidence states (19%) than what is typically seen in public health surveillance (≈5%).

Our findings underscore the large clinical burden associated with Lyme disease diagnoses in the United States. Evolving electronic medical and laboratory systems should help fill demonstrable data gaps and enable more robust and reliable monitoring of changes in the magnitude and spread of the disease. Effective interventions are needed, and improved awareness among clinicians and the public is paramount to foster early and accurate diagnosis and appropriate treatment.
